# Acoustic analysis of clients’ expression of self-compassion, self-criticism, and self-protection within emotion focused therapy video sessions

**DOI:** 10.3389/fpsyg.2024.1363988

**Published:** 2024-04-23

**Authors:** Ghazaleh Bailey, Júlia Halamová, Viktória Vráblová

**Affiliations:** Institute of Applied Psychology, Faculty of Social and Economic Sciences, Comenius University in Bratislava, Bratislava, Slovakia

**Keywords:** emotion focused therapy, pitch, intensity, acoustic analysis, Praat

## Abstract

**Introduction:**

When it comes to the non-verbal communication of emotions, it is apparent that the human voice is one of the main ways of expressing emotion and is increasingly important in psychotherapeutic dialog. There is ample research focusing on the vocal expression of emotions. However, to date the analysis of the vocal quality of clients’ in-sessional emotional experience remains largely unexplored. Moreover, there is generally a gap within the psychotherapy literature in the understanding of the vocal character of self-compassion, self-criticism, and protective anger.

**Methods:**

In this study we investigated how clients vocally convey self-compassion, self-protection and self-criticism in Emotion Focused therapy sessions. For this purpose we investigated 12 commercially available Emotion Focused Therapy videos that employed a two chair or empty chair dialog. Praat software was used for the acoustic analysis of the most common features – pitch (known as fundamental frequency or F0) and intensity (voice amplitude, i.e., loudness).

**Results:**

Results showed that intensity was significantly higher for self-criticism and self-protection than for self-compassion. Regarding pitch the findings showed no significant differences between the three states.

**Discussion:**

More research analyzing acoustic features in a larger number of cases is required to obtain a deeper understanding of clients’ vocal expression of self-compassion, self-protection and self-criticism in Emotion Focused Therapy.

## Introduction

In Emotion Focused Therapy (EFT) emotional processing is acknowledged to have significance for therapeutic change (Pascual-Leone and Greenberg, 2007; Auszra et al., 2013; Pascual-Leone, 2017; Haberman et al., 2018). According to the EFT model of change, self-protection (also known as assertive anger) and self-compassion are particularly relevant to the transformation of self-criticism (Pascual-Leone and Greenberg, 2007; Timulak, 2015; Pascual-Leone, 2017). Furthermore, it is well known that self-criticism is associated with high levels of shame and negative self-evaluation (Blatt and Zuroff, 1992; Gilbert et al., 2004; Haberman et al., 2018), and that the activation of self-compassion and self-protection are recognized as effective responses for coping with self-criticism (Pascual-Leone, 2017; Halamová et al., 2018, 2019; Halamová and Kanovský, 2019). In EFT it is further established that developing self-compassion and self-protection are substantial steps to transforming global distress (e.g., hopelessness, self-pity, helplessness, guilt) (Timulak, 2015; Pascual-Leone, 2017). Therefore, in the sequential emotional processing model (for more information see Pascual-Leone and Greenberg, 2007; Pascual-Leone, 2017) self-compassion and self-protection are seen as primary adaptive emotions and key elements to the process of emotional change. Expressing unmet needs through self-protection, being kind, caring and acknowledging human imperfection through self-compassion strengthens the self and decreases negative self-evaluation and self-criticism (Greenberg and Watson, 2006; Greenberg, 2015; Timulak, 2015; Singh et al., 2020). One of the major ways in which EFT therapists help clients transform maladaptive emotions into primary adaptive emotions is through two chair enactments. EFT distinguishes between two sorts of two chair dialogs: self–self dialogs with specific parts of the self (e.g., critical voice) and empty chair dialogs with imaginary significant others (Greenberg and Malcolm, 2002; Shahar et al., 2012; Goldman, 2017). In self-critical chair work the therapist facilitates a conversation between the critical part of the client and the experiencing self who receives the criticism (e.g., Shahar et al., 2012). During an empty chair enactment the therapist encourages the client to express unfinished, problematic emotional experiences to a significant other and work through them (Greenberg and Malcolm, 2002). Both two chair techniques are aimed at promoting clients’ abilities to be compassionate toward their painful emotional experiences and developing self-protection capacities. Therefore, whether these adaptive emotions are expressed toward the self in a two chair dialog or in an empty chair dialog with a significant other makes little difference. Although activation of self-criticism, self-compassion and self-protection are acknowledged as being essential for the emotional change process in EFT, how these states are expressed in EFT sessions is a grey area. Clients express these states through different types of verbal as well as nonverbal communication including vocal quality.

When it comes to the non-verbal communication of emotions, it is apparent that the human voice is becoming increasingly important in psychotherapeutic dialog (Rice and Kerr, 1986; Tomicic and Martínez Guzmán, 2011) and one of the main ways in which emotions are expressed. There is ample research investigating the vocal expression of emotion (Kappas et al., 1991; Bachorowski and Owren, 1995; Bachorowski, 1999; Scherer, 2003; Scherer et al., 2003, 2011; Simon-Thomas et al., 2009) and demonstrating the relationship between emotional state and the acoustic characteristics of vocalizations (Kappas et al., 1991; Bachorowski and Owren, 1995; Banse and Scherer, 1996; Scherer, 2003; Scherer et al., 2003). In the literature this is called the vocal expression of emotion (Kappas et al., 1991; Bachorowski and Owren, 1995; Banse and Scherer, 1996). Generally research on the vocal expression of emotion lags someway behind the study of facial affect expression (Bailey et al., 2023). Until now research in the field of emotion recognition in speech has been mainly based on laboratory acted emotions, examining sustained vowels (Scherer et al., 1991; Banse and Scherer, 1996; Bachorowski and Owren, 2001) or utterances (e.g., Razak et al., 2003). Fewer studies have been published that use naturally occurring speech (e.g., Crangle et al., 2019). According to Parsa and Jamieson (2001) there are only a small number of studies investigating acoustic features in running speech and to our best knowledge there has been no paper evaluating pitch and intensity in continuous speech in therapy sessions. Rochman et al. (2008) were among the first to examine the vocal quality of women expressing sadness and unresolved anger in a psychotherapy setting using a two chair dialog with an attachment figure. They demonstrated the value of computerized acoustic analysis in recognizing clients’ productive expression of emotional experiences. Recent advances in machine learning permit researchers to analyze vocal affect expression automatically (Vogt et al., 2008; Rabiei and Gasparetto, 2015; Kumar and Iqbal, 2019). Computerized speech recognition allows the tracking of emotion in speech in more natural settings.

Based on the present state of the evidence, the consensus is that the vocal expression of emotion involves the following variables (Kappas et al., 1991; Bachorowski and Owren, 1995; Banse and Scherer, 1996): level of frequency (perceived pitch of a sound, referred to as F0), amplitude (voice intensity, e.g., loudness), voice quality (e.g., breathing, hoarseness, harshness) and duration (speech rate, e.g., tempo and pausing). However, the most commonly analyzed features in emotion recognition [e.g., in Scherer et al. (2003), Magdin et al. (2019)] are: fundamental frequency (F0, perceived as pitch), intensity (perceived as loudness), jitter (pitch perturbation) and shimmer (intensity perturbation). Dynamic changes in pitch and intensity have been related to emotional arousal: for instance, anger is identified with a high pitch and high intensity whereas sadness is identified with a lower pitch and low intensity (Scherer et al., 2003; Rochman et al., 2008). Up until now the research has mainly focused on the analysis of basic emotions such as anger, sadness, happiness and fear (Kappas et al., 1991; Banse and Scherer, 1996; Scherer, 2003).

Consequently, there is a gap in the understanding of the vocal character of self-compassion, self-criticism and self-protection. This study was designed to identify the acoustic features, pitch and intensity of self-compassion, self-criticism and self-protection through the computer analysis of the speech signals of clients in EFT therapy sessions in a two chair or empty chair dialog. First, we review the current state of the research on the vocal analysis of self-compassion, self-criticism and self-protection generally.

### Vocal expression of self-criticism

Although the importance of self-critical talk is well known in research and practice, there is a gap in the literature on the acoustic analysis of self-criticism. While there is ample research on the verbal expression of self-criticism (Whelton and Henkelman, 2002; Whelton and Greenberg, 2005; Kannan and Levitt, 2013), there is little knowledge on what self-criticism sounds like in practice when articulated by clients in therapy sessions. To the best of our knowledge and to date there has been no study examining the vocal expression of self-criticism. However, in EFT theory self-criticism is considered to be a type of problematic anger and viewed as a secondary emotion (Pascual-Leone et al., 2013; Kramer and Pascual-Leone, 2015). In the EFT model secondary emotions are reactive, defensive responses to a primary emotion (Pascual-Leone et al., 2013; Herrmann et al., 2016). Thus, self-criticism (as problematic anger) is defined as a secondary emotion to the primary maladaptive emotion of shame, characterized as the expression of self-hate and contempt by highly critical people (Blatt and Zuroff, 1992; Whelton and Greenberg, 2005; Kannan and Levitt, 2013). Furthermore, Beuchat et al. (2023) describe self-contempt as the fundamental emotion behind self-criticism signifying anger and disgust toward oneself. According to Kramer and Pascual-Leone (2015) people who are vulnerable to maladaptive anger express more self-contempt when criticizing themselves. Moreover, their study demonstrates the importance of the tone participants use when being self-critical, as a major factor affecting level of self-criticism. Hence, self-criticism is associated with the expression of anger toward oneself (Luyten and Blatt, 2012; Abi-Habib and Luyten, 2013; Pascual-Leone et al., 2013; Kramer and Pascual-Leone, 2015). In addition, according to the classification of affective meaning states (CAMS; Pascual-Leone and Greenberg, 2005) the anger known as “rejecting anger” is characterized by an increase in pitch and moderate to high intensity. We therefore assume that the voice quality of self-criticism is similar to that of rejecting anger. Rochman et al. (2008) were among the first to investigate the vocal quality of maladaptive anger in a therapeutic setting by analyzing the expression of anger before and after expressing sadness. Their findings are in line with previous research demonstrating an increase in pitch (Breitenstein et al., 2001; Razak et al., 2003; Yildirim et al., 2004) and intensity (Kapoor and Sagar Verma, 2019) associated with anger. Furthermore, it has been established that higher activated emotions (e.g., anger and happiness) are linked to higher fundamental frequency (F0) (Breitenstein et al., 2001) and voice intensity (Schröder et al., 2001).

### Vocal expression of self-compassion

In contrast to the situation regarding self-criticism, there has been greater interest over the last few years in examining and differentiating a broader range of emotions such as a variety of positive emotions like compassion (Simon-Thomas et al., 2009; Kamiloğlu et al., 2020). In their review of vocal expressions of positive emotions, Kamiloğlu et al. (2020) systematically compared 108 studies investigating acoustic features across different positive emotions, highlighting differences in pitch, loudness, and speech rate. The authors classified positive emotions into emotion families such as epistemological emotions (amusement, interest, relief), savoring emotions (contentment and pleasure) and prosocial emotions (admiration). According to that study compassion is categorized as a positive emotion in connection with, for instance, kindness, contentment, pleasure and gratitude. This group of low aroused positive emotions contrasts with epistemological emotions described as having moderate pitch and loudness and a slower speech rate. The low level of these emotions characterizes their purpose: “adaptive functions for the person experiencing them” (Kamiloğlu et al., 2020, p.24). Furthermore, Simon-Thomas et al. (2009) indicated that compassion is communicated through vocal bursts. They investigated vocal bursts of 22 emotions. In their study judges were asked to listen and identify different vocal bursts of emotions expressed by actors. “Posers” were asked to express 22 emotions without using words. Compassion was grouped as a positive, pro-social emotion along with contentment, amusement, gratitude, love and so forth, and were identified accurately. The judges identified compassion correctly in 30% of the positive vocal bursts and in 47% of the selected, prototypical vocal bursts. In line with Kamiloğlu et al. (2020) self-compassion is defined in EFT as a primary adaptive emotion that helps clients to be caring and attentive to their needs (Pascual-Leone, 2017). Diamond et al. (2010) were among the first to examine the acoustic parameters of primary emotions in an EFT setting. Their findings are in line with prior research (Scherer et al., 2003) and show a decrease in pitch and intensity during the expression of sadness when attending to one’s losses, in comparison to non-emotional speech. According to Neff (2003) and Strauss et al. (2016) self-compassion is compassion directed toward oneself. It is defined as self-kindness and the ability to turn to and feel empathy for one’s own suffering, among other things. Consistent with this Pascual-Leone and Greenberg (2005) describe self-compassion as the presence of caring, tenderness and nurturing by the self through a variety of self-compassion dialogs such as caring for the inner child or a significant other, offering soothing in an imaginary dialog. For this reason, in this study the expression of compassion and self-compassion are understood to be equivalents. Taken together these studies show compassion is identified as a low aroused adaptive positive emotion (Simon-Thomas et al., 2009; Kamiloğlu et al., 2020) marked by a moderate pitch and intensity similar to love, kindness (Sauter, 2017; Kamiloğlu et al., 2020).

### Vocal expression of self-protection

In contrast to maladaptive anger expressed through self-criticism, arousing self-protection as primary adaptive anger is one of the key aspects of emotional change in EFT and a core factor in treatment outcomes (Greenberg and Malcolm, 2002; Pascual-Leone, 2017). Helping clients to stand up and express their unmet needs in an assertive manner strengthens their self and increases their sense of worthiness (Pascual-Leone and Greenberg, 2007; Timulak, 2015). Therefore, self-protection contrasts to rejecting anger, particularly when characterized by a sense of entitlement and positive self-affirmation (Pascual-Leone and Greenberg, 2005; Timulak, 2015; Pascual-Leone, 2017). As stated in CAMS (Pascual-Leone and Greenberg, 2005) self-protection is experienced with a moderate to high expressive arousal and loud voice. The increased sense of agency and empowerment is expressed in the anger. Unlike when expressing rejecting anger, a client expressing self-protection “is strong, clear and well-grounded, and speaks with a sense of growing confidence” (Pascual-Leone and Greenberg, 2005, p. 57). There has been no study on the acoustic parameters of this type of anger comparing it to rejecting anger.

## Aim of the study

To our best knowledge there has been no study on the acoustic analysis of self-compassion, self-protection and self-criticism in running speech in psychotherapy sessions. Therefore, the purpose of this study was to shed light on the following *research question*: What are the characteristic acoustic features of pitch and intensity in clients’ expression of self-compassion, self-protection and self-criticism in real EFT-therapy sessions?

### Hypothesis 1

Based on previous findings and self-criticism defined as a form of rejecting anger, we hypothesize that clients expressing self-criticism will have significantly higher pitch (e.g., Breitenstein et al., 2001; Razak et al., 2003; Yildirim et al., 2004) and intensity (Kapoor and Sagar Verma, 2019) in comparison to baseline-controlled self-compassion.

### Hypothesis 2

Considering the adaptive functions of self-compassion together with previous research defining compassion as a low aroused positive emotion (e.g., Kamiloğlu et al., 2020), we expect that clients’ expressions of self-compassion in an EFT session will have significantly lower pitch and intensity in comparison to self-criticism, and self-protection controlled for baseline.

### Research question 1

As there is no research on the vocal quality of self-protection, we want to deepen our understanding by examining the following question: How do the acoustic parameters of the pitch and intensity of self-protection differ from self-compassion and self-criticism controlled for their baseline?

## Methods

### Materials

For the purpose of this study we decided to examine previously recorded videos of real EFT therapy sessions that are commercially available and can be used for research purposes. The following criteria were set for selecting the videos for this analysis. The videos had to be in English, show clients expressing self-compassion, self-protection or self-criticism in a two chair or empty chair dialog in a therapy session, and the voice quality of the tapes had to be sufficiently good for computer software recognition. A total of 17 EFT sessions were reviewed and 12 were identified as valid examples based on the first author’s requirements and consulted with the second author. Four sessions were two chair dialogs with the critical self and eight were empty chair dialogs with a significant other. The therapy sessions were led by EFT experts. All the clients were female.

*The EFT videos were on the following topics:*

Leslie Greenberg as therapist:

- *EFT over time with Marcy*. Psychotherapy in six sessions (American Psychological Association, 2007a,b). Sessions 2, 3, 4, and 6 were selected for this study.
- EFT for Depression with Dione (American Psychological Association, 2007a).
  This is a series of two sessions with Dione who is suffering from depression. Both sessions were chosen for the study.
- Working with cor emotion with Darum (CPCAB, 2020a).
- Working with current and historical trauma with Sam (CPCAB, 2020b).
- Working with social anxiety with Dawn (Psychological and Educational Films, 1989).

*Sandra Paivio as therapist:*

- Narrative processes in EFT with Hannah (American Psychological Association, 2015).

*Rhonda Goldman as therapist:*

- Case formulation in EFT. Addressing unfinished business with Candy (American Psychological Association, 2014).

*Ladislav Timulak as therapist:*

- Transforming Emotional Pain: An Illustration of Emotion-Focused Therapy video with Claire (CPCAB, 2020c).

### Procedure

Voice sections segmenting self-compassion, self-criticism and self-protection were coded and extracted from each 50-min therapy session by the first author and reviewed by the second author. Both coders were certified EFT therapists and trained to recognize the states of self-compassion, self-criticism and protective anger. The selected sequences were converted into .wav files with VLC (version 3.0.12, VideoLan, 2006). Extracts of the therapist’s voice were trimmed using audacity (Version 2.3.3; R Core Team, 2019), a free audio editing tool. There are several different technical challenges involved in recognizing emotion in naturally occurring running speech in previously recorded videos. The most challenging aspects include the microphone and the environmental conditions. Our videos were not specially recorded for acoustic analysis and so the voice recordings were not of the required quality. As the recordings were poor quality and to increase the pitch determination algorithm accuracy of Praat, a profile of the background noises was obtained from the silences in each audio segment. Then the background noise was reduced, using the frequencies of the noises previously obtained. In order to eliminate the pitch-outliers and enhance the accuracy of the analysis, the outliers in maximum and minimum pitches were eliminated using the spectrum analysis in the Praat software. The software mistakenly identified most of these outliers as voice pitch. In reality though most were caused by background noises or other sources and were not emitted by the client. The final audio versions were then used for the feature extractions in Praat (Version 6.1.34; Boersma and Weenink, 2018). In the end, the length of the self-criticism sequences was between 0:28 min and 01:34 min; the self-compassion sequences were between 0:25 min and 03:26 min; and self-protection between 01:02 min and 04:35 min. Altogether there were seven clients and 06:84 min of self-criticism, ten clients and 11:76 min of self-compassion and ten clients and 15:19 min of protective anger. According to Diamond et al. (2010, p. 405) at least “three sentences of speech are necessary for emergent emotions to be sufficiently formed.” All the audios in our study fulfilled this requirement. The baseline was extracted from 0:30 to 02:30 min of each session. We decided to start from 0:30 min as in the majority of the videos the therapists started the sessions with a short introduction about EFT and the clients had not begun speaking. The time sequence for the baseline at the beginning of the session was chosen to capture he clients’ emotional neutral speech. We followed a similar approach to Rochman et al. (2008) with the timing differing as described above. Moreover, according to previous research (Watson and Bedard, 2006; Auszra et al., 2013) clients’ level of emotional processing increases throughout the session and is at its lowest at the beginning of the session before the working phase. In view of this, the segments selected for the baseline were the most ideal in terms of emotional neutral speech. After extracting the client’s voice a minimum of 0:40 and maximum of 01:16 min remained for the baseline, totaling 8:45 min for all 12 sessions.

### Measurement instrument

Praat is among the most commonly used software for computerized acoustic analysis (Boersma and Weenink, 2018). Developed by Boersma and Weenink, Praat is a free program for the analysis and reconstruction of acoustic speech signals and available for all major computer platforms (Boersma, 2013b). It offers a wide range of procedures (Boersma and Weenink, 2018) relevant to speech emotion including fundamental frequency (pitch), speech rate, pauses, voice intensity, jitter (pitch perturbations) and shimmer (loudness perturbations). As mentioned above the vocal expression of emotion is characterized by several acoustic features (Bachorowski and Owren, 1995; Banse and Scherer, 1996). For the purpose of this study we decided to examine the most commonly used prosodic parameters that effectively characterize emotion in running speech and can be directly measured by the software (Boersma, 2013a). We decided to set the time step strategy to automatic as recommended by Praat. That way Praat computes just enough pitch and intensity values to draw reliable pitch and intensity contours (Boersma and Weenink, 2003).

#### Fundamental frequency (F0)

The human voice produces sounds through vocal fold vibration and resonance. This vibration produces the sound wave of the voice (Chen, 2016). The rate of the vocal fold vibrations determines the fundamental frequency F0 of the voice and is measured in Herz (Hz). Cyclic variations in the fundamental frequency are recognized by the listener as pitch. The higher the frequency of the vocal folds vibrations, the higher the pitch (Chen, 2016; Dasgupta, 2017). As reviewed by Razak et al. (2003) the pitch contour of a speaker is the most valuable indicator of the person’s emotional state. Women’s vocal folds, for instance, vibrate faster than men’s. That is why female voices have a higher mean pitch than male voices (Murray and Arnott, 1993). According to the literature the average pitch range for women is 100–300 Hz (Traunmüller and Eriksson, 1994; Rochman, 2008). In their literature review on human vocal emotion, Murray and Arnott (1993) state that shouting not only displays an increase in intensity but also in pitch, while tiredness and sadness are characterized by a lower pitch, compared to neutral speech. Based on an acoustic analysis of emotions in speech (Yildirim et al., 2004) the pitch range for sadness is between 66 Hz and 195 Hz and the average pitch of neutral emotion is between 49 Hz and188 Hz. Furthermore, Yildirim et al. (2004) report that aroused emotions such as anger and happiness are linked to even higher pitch values than are sadness and emotional neutral speech. The pitch range for anger is around 140–400 Hz (Razak et al., 2003; Kapoor and Sagar Verma, 2019) and the average pitch for happiness is over 176 Hz (Razak et al., 2003; Yildirim et al., 2004). Self-compassion is often related to sadness and calmness. These emotions are often characterized by a lower pitch, whereas self-criticism is defined as a form of rejecting anger, which is associated with higher pitch values. Given that all our clients were female and based on previous studies (Rochman, 2008), the pitch range in Praat was set at 100–300 Hz.

#### Intensity (amplitude)

The intensity of the sound is defined as the loudness of the sound. The amplitude of the vibrations (i.e., the size of the oscillations of the vocal folds) affects the loudness. The loudness depends on the level of air pressure in the lungs. The higher the intensity, the louder the voice. The sound intensity is measured in decibels (dBs). The standard intensity setting in Praat is from 50 dB to 100 dB. Sadness is associated with a lower intensity (between 60 dB and 68 dB) and anger with a higher intensity (max. 85 dB) (Kapoor and Sagar Verma, 2019) than emotional neutral speech.

### Data analysis

For statistical purposes, we used program R version 4.0.2 (R Core Team, 2019). Our measures were repeated within individuals. We therefore used package “lme4” (Bates et al., 2015) to fit a multilevel model (8 respondents in total, and 3 states for 2 vocal expressions for each respondent). Two vocal expressions included pitch and intensity in 3 states – self-criticism, self-compassion and self-protection. We decided to do two separate multilevel models because vocal expression pitch was measured in Hz and intensity was measured in dB. The parameters of the first multilevel model were ID (variability among respondents) and Pitch (variability among the pitch states mentioned above). For the second multilevel model the parameters were ID (variability among respondents) and Intensity (variability among intensity states). We treated these as random effects and used a logistic multilevel regression model. In this study we report conditional R^2^ measure (the overall effect size) and random effects variance (ID, Pitch and ID, Intensity).

## Results

**Figure 1:**
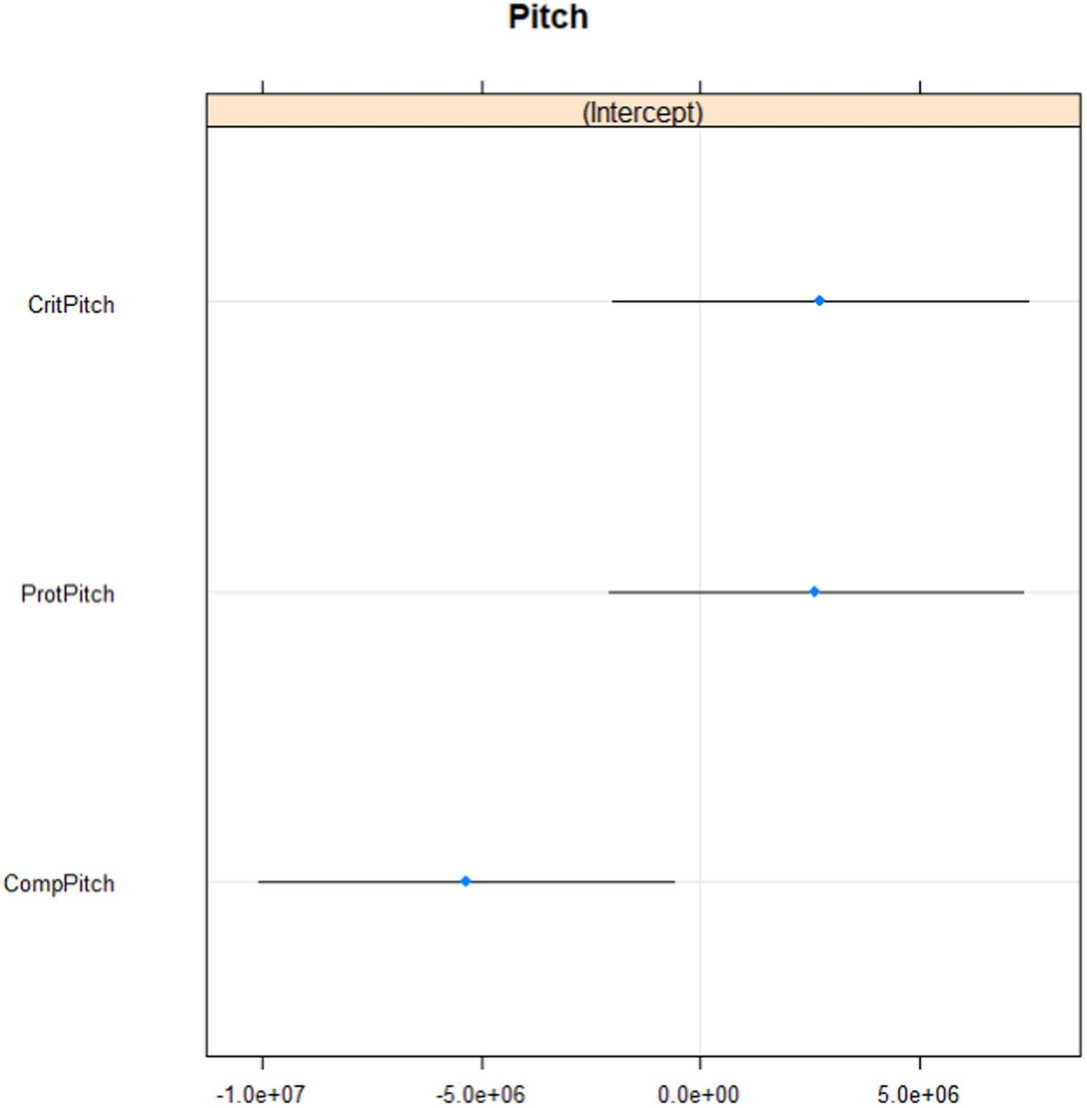
Pitch during self-criticism, self-compassion and self-protection. Prot = self-protection. Crit = self-criticism. Comp = self-compassion.

**Figure 2:**
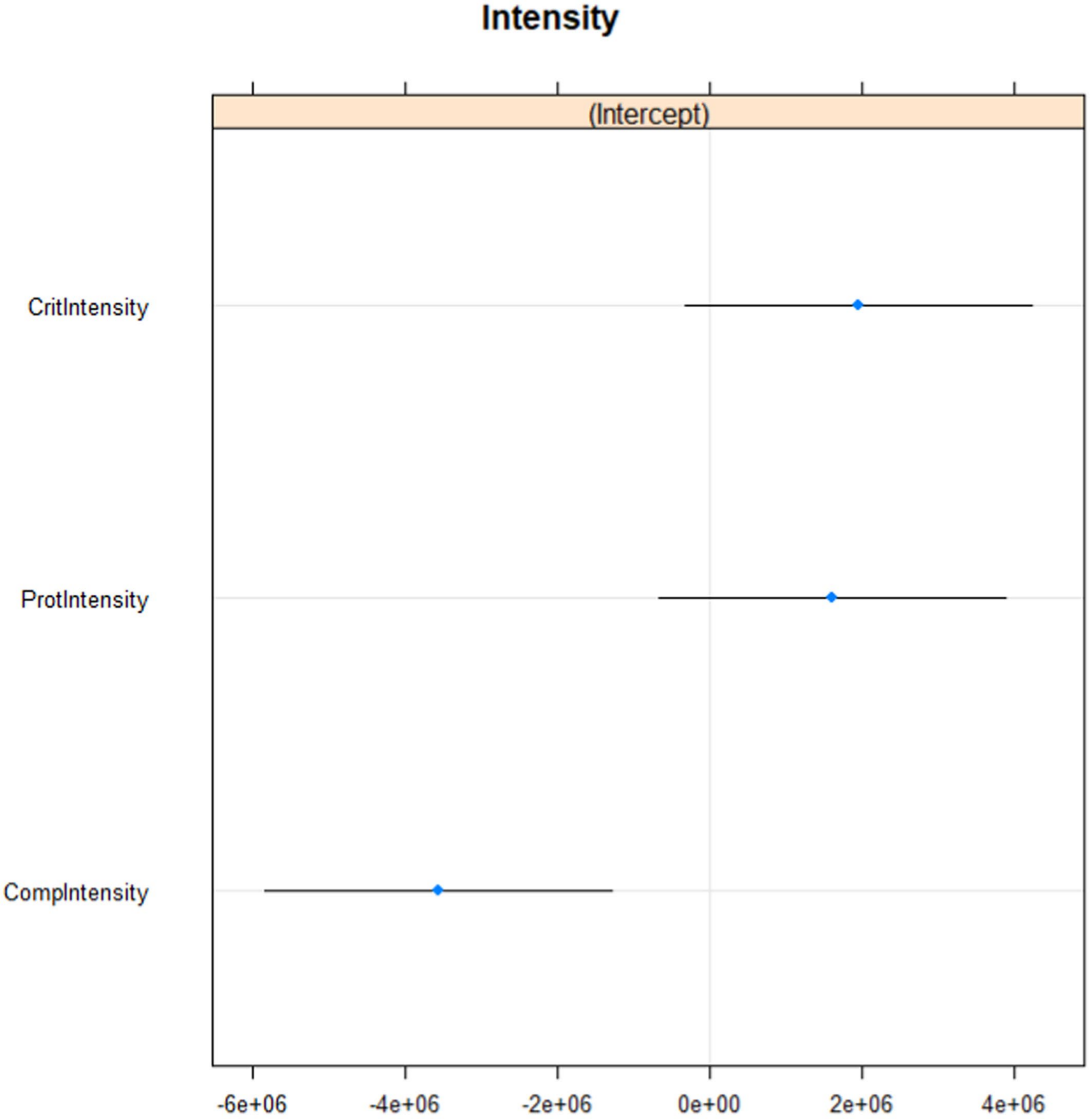
Intensity during self-criticism, self-compassion and self-protection.

Baseline mean for Pitch was 179.57 and for Intensity 61.24. The number of observations for Pitch was 115,097 and the number of observations for Intensity was 274,171. The ID variance for the first multilevel model was 3.061e+14, and Pitch variance was 2.146e+13, which means that respondent variance was far larger than Pitch variance (individual differences between respondents were larger than differences among pitch states). *R*^2^ for the first model was 0.15. The ID variance for the second multilevel model was 2.080e+13 and the Intensity variance was 9.567e+12. *R*^2^ for the second model was 0.10. All Intensity states had lower means than the baseline mean. Intensity for self-criticism and self-protection was significantly higher than for self-compassion. See Figures 1, 2 for details of the vocal expressions – pitch and intensity.

## Discussion

The aim of this study was to examine the pitch and intensity features of self-compassion, self-criticism and self-protection in real EFT-therapy sessions. The first hypothesis was that clients expressing self-criticism would show an increase in pitch and intensity in comparison to self-compassion. Our results confirmed higher values for intensity of self-criticism compared to self-compassion, but not for higher pitch. Within the literature self-criticism is acknowledged to be a form of reactive anger (Pascual-Leone et al., 2013) and self-compassion a positive, low aroused emotion (Kamiloğlu et al., 2020). Thus, the values are in line with previous research identifying higher intensity values for anger (Banse and Scherer, 1996), and not for compassion (Kamiloğlu et al., 2020), which is a low aroused emotion. As a high aroused emotion anger is characterized through high levels of intensity (Banse and Scherer, 1996; Sobin and Alpert, 1999; Rochman et al., 2008; Kapoor and Sagar Verma, 2019). This is in agreement with previous findings characterizing physiological arousal with higher levels of pitch and intensity (Banse and Scherer, 1996). Thus, when clients start criticizing themselves, they become emotionally activated and their voices get louder. It is probable that if there had been more participants in the analysis, significance would also have been yielded for pitch, not just intensity, of self-criticism. However, this outcome is not in line with our prediction about pitch. Self-criticism shows lower intensity values than the baseline (emotional neutral speech). Overall, the results indicate lower values for all states in comparison to the baseline. One reason for this could be the technical environment. Our voice samples were extracted from videos that did not meet laboratory conditions. As a result, the microphone setting could not be adjusted to obtain a suitable speech recording, which had that been possible may have led to different values than assumed. However, though scholars agree that activated emotions such as reactive anger have higher intensity levels (e.g., Banse and Scherer, 1996; Sobin and Alpert, 1999), clients do not appear to express their anger as loudly as actors do when asked to elicit emotions (e.g., Banse and Scherer, 1996). According to Sobin and Alpert (1999) emotions expressed by actors do not fully capture the complexity of emotions. As a consequence, it is unclear to what extent actors are able to represent emotions as they would naturally be expressed by human beings. In this study, our results demonstrate that the clients did not become more activated but used emotionally neutral speech in the therapy session. Nevertheless, there were differences between the states. In line with our second prediction, self-compassion had lower intensity values than both self-protection and self-criticism, but this did not apply to pitch. Self-protection had higher intensity values than self-compassion and lower intensity values than self-criticism. However, only the difference between self-compassion and self-protection is significant. This outcome fits the definition that self-protection is a form of healthy, protective anger (Pascual-Leone and Paivio, 2013; Pascual-Leone, 2017), characterized through firmness and strength (Timulak, 2015). So far there has been no study on the vocal expression of self-protection. Our results suggest that when clients feel powerful their voices increase strongly and the intensity rises. The results on the pitch values are not in line with our assumptions. However, the findings are not significant enough to allow accurate interpretation. From our findings we can conclude that emotional neutral speech has lower pitch values than self-compassion, self-protection and self-criticism. The findings for self-protection and self-criticism are in accordance with previous research showing higher pitch values for higher aroused emotions such as anger (Razak et al., 2003; Yildirim et al., 2004). Self-compassion is a low aroused emotion and earlier studies report lower values for it than for emotional neutral speech (Razak et al., 2003; Yildirim et al., 2004). But we cannot validate this in our paper. Pitch values can differ between individuals for various reasons. All our clients were female and were speaking English in the sessions, but pitch values may also depend on sexual orientation (Waksler, 2001), first language (Andreeva et al., 2014) or cultural background for instance (Scherer et al., 2011). As mentioned above (see procedure), part of the problem in demonstrating significant differences between self-compassion, self-protection and self-criticism is the challenge of measuring pitch within continuous speech in natural settings. Another important factor that can lead to a poor outcome is the low number of voice samples analyzed. Moreover, the clients in the selected audios represent different kinds of problematic behaviors. As Rochman et al. (2008) point out it is important to note that different psychological disorders, such as depression or anxiety, can influence the way emotions are vocally expressed. It is worth mentioning that, so far, the majority of the research has focused on the investigation of intensity and pitch values in acted laboratory settings. This paper is the first to examine these features in spontaneous, continuous speech in an EFT therapy session.

### Limitations

Our research limitations are as follows. First, it is important to point out that our audio material was not of a high recording quality. Therefore, we did not have the required voice quality for the Praat feature analysis, although the background noises were eliminated. Second, the segments for self-compassion, self-protection and self-criticism were trimmed from different points in the therapy. According to Nardone et al. (2021) there is a significant connection between the temporal sequence of expressed emotional arousal, the vocalization of unmet needs and treatment outcome. Similarly, Rochman et al. (2008) demonstrated the sequence of expressing emotions has an impact on the acoustic articulation of the emotion. As we extracted the analyzed sequences throughout the sessions, we could not ensure how the timing might have had an influence on the pitch and intensity values. Furthermore, as not all clients were expressing self-compassion, self-protection and self-criticism we had a different number of clients for each state. Thus, our material was not consistent. In addition, two clients were analyzed twice, as they appeared in multiple therapy sessions. This, along with the fact that the length of the audios varied owing to the natural setting, meant we had no control over how long the clients expressed each state in the session. Also, although our aim was to examine pitch and intensity features in real therapy sessions, the sessions were studio recorded so we could not ensure that the setting was completely natural. Lastly, it is important to mention the low number of clients voice extracts and the fact that all the clients were female and were speaking English. Our findings therefore tell us little about the pitch and intensity of these states in men or other languages.

### Further research

Future research should be conducted in more appropriate technical conditions. The microphone needs to be set up so that it consistently captures the voice, and a quiet setting should be used. In order to ensure that emotionally neutral speech is used as the baseline we suggest extracting clients voices from a natural conversation before the therapy session. We also recommend analyzing the same number of voice extracts for each state and, insofar as is possible in natural settings, making sure that all three states are expressed by each client. It goes without saying that these things are of course challenging to achieve in real therapy sessions. A larger number of clients voice samples would also yield a higher number of values, which could lead to a more differentiating outcome. Future studies could investigate additional acoustic features such as speech rate, harmonicity and perturbations, and could examine dynamic changes in the features throughout the therapy session. Finally, researchers in this field could examine gender, culture and language differences in self-compassion, self-protection and self-criticism in real EFT therapy sessions.

## Conclusion

Our research analyzing the pitch and intensity of self-compassion, self-protection and self-criticism in EFT therapy videos is a first step toward investigating acoustic features of clients in real psychotherapy sessions. While earlier research has focused on the analysis of vowels or sustained vocals, we examined the pitch and intensity of self-compassion, self-protection and self-criticism in running speech using Praat computer software. Our results demonstrate that whereas self-criticism had the highest intensity values, self-compassion had the lowest among the three states. Our findings on pitch were not significant.

## Data availability statement

The original contributions presented in the study are included in the article/supplementary material, further inquiries can be directed to the corresponding author.

## Ethics statement

The studies involving humans were approved by Ethical committee of Faculty of social and economic sciences Comenius University. The studies were conducted in accordance with the local legislation and institutional requirements. The participants provided their written informed consent to participate in this study.

## Author contributions

GB: Conceptualization, Data curation, Formal analysis, Project administration, Software, Writing – original draft, Writing – review & editing. JH: Conceptualization, Funding acquisition, Methodology, Resources, Supervision, Validation, Writing – review & editing. VV: Formal analysis, Software, Writing – review & editing.

## References

[ref1] Abi-Habib R. Luyten P. (2013). The role of dependency and self-criticism in the relationship between anger and depression. Personal. Individ. Differ. 55, 921–925. doi: 10.1016/j.paid.2013.07.466

[ref2] American Psychological Association. (2007a). Emotion-focused therapy for depression. Available at: https://www.apa.org/pubs/videos/4310798?tab=1.

[ref3] American Psychological Association. (2007b). Emotion-focused therapy over time. Psychotherapy in six sessions video series. Available at: https://www.apa.org/pubs/videos/4310761

[ref4] American Psychological Association. (2014). Case formulation in emotion-focused therapy. Available at: https://www.apa.org/pubs/videos/4310916.

[ref5] American Psychological Association. (2015). Narrative processes in emotion-focused therapy for trauma. Available at: https://www.apa.org/pubs/videos/4310940.

[ref6] Andreeva B. Demenko G. Wolska M. Möbius B. Zimmerer F. Jügler J.. (2014). Comparison of pitch range and pitch variation in Slavic and Germanic languages. Proc. Int. Conf. Speech Pros. 776–780. doi: 10.21437/speechprosody.2014-143

[ref7] Auszra L. Greenberg L. Herrmann I. (2013). Client emotional productivity-optimal client in-session emotional processing in experiential therapy. Psychother. Res. 23, 732–746. doi: 10.1080/10503307.2013.816882, PMID: 23848974

[ref8] Bachorowski J. A. (1999). Vocal expression and perception of emotion. Curr. Dir. Psychol. Sci. 8, 53–57. doi: 10.1111/1467-8721.00013

[ref9] Bachorowski J. A. Owren M. J. (1995). Vocal expression of emotion: acoustic properties of speech are associates with emotional intensity and context. Psychol. Sci. 6, 219–224. doi: 10.1111/j.1467-9280.1995.tb00596.x

[ref10] Bachorowski J. A. Owren M. J. (2001). Not all laughs are alike: voiced but not unvoiced laughter readily elicits positive affect. Psychol. Sci. 12, 252–257. doi: 10.1111/1467-9280.00346, PMID: 11437310

[ref11] Bailey G. Halamová J. Vráblová V. (2023). Clients’ facial expressions of self-compassion, self-criticism, and self-protection in emo. Int. J. Environ. Res. Public Health 20, 1–14. doi: 10.3390/ijerph20021129 PMC9859613 36673885

[ref12] Banse R. Scherer K. R. (1996). Acoustic profiles in vocal emotion expression. J. Pers. Soc. Psychol. 70, 614–636. doi: 10.1037/0022-3514.70.3.614 8851745

[ref9001] Bates D. Maechler M. Bolker B. Walker S. (2015). Fitting Linear Mixed-Effects Models Using lme4. Journal of Statistical Software, 67, 1–48.

[ref13] Beuchat H. Grandjean L. Junod N. Despland J. N. Pascual-Leone A. Martin-Sölch C.. (2023). Evaluation of expressed self-contempt in psychotherapy: an exploratory study. Couns. Psychol. Q. 179457, 1–16. doi: 10.1080/09515070.2023.2201417

[ref14] Blatt S. J. Zuroff D. (1992). Interpersonal relatedness and self-definition: two prototypes for depression from an ethological and object relations point of view. Clin. Psychol. Rev. Psychol. Reuim 12, 527–562. doi: 10.1016/0272-7358(92)90070-O

[ref15] Boersma P. (2013a). “Acoustic analysis” in Research methods in linguistics. eds. Podesva R. Sharma D. (Cambridge: Cambridge University Press)

[ref16] Boersma P. (2013b). “The use of Praat in corpus research” in The Oxford handbook of corpus phonology. eds. Durand J. Gut U. Kristoffersen G. (Oxford: Oxford University Press)

[ref17] Boersma P. Weenink D. (2018). Praat: doing phonetics by computer (p. version 6.1.34). Available at: https://www.fon.hum.uva.nl/praat/download_mac.html

[ref18] Boersma P. Weenink D. (2003). Time step settings. Available at: https://www.fon.hum.uva.nl/praat/manual/Time_step_settings___.html

[ref19] Breitenstein C. Van Lancker D. Daum I. (2001). The contribution of speech rate and pitch variation to the perception of vocal emotions in a German and an American sample. Cognit. Emot. 15, 57–79. doi: 10.1080/0269993004200114

[ref20] Chen C. J. (2016). Elements of human voice Singapore: World Scientific publishing co.

[ref21] CPCAB. (2020a). Leslie Greenberg: working with core emotion. Available at: https://www.cpcab.co.uk/shop/les-greenberg-wwce-video.

[ref22] CPCAB. (2020b). Leslie Greenberg: working with current and historical trauma. Available at: https://www.cpcab.co.uk/shop/les-greenberg-wwcht-video.

[ref23] CPCAB. (2020c). Transforming emotional pain: an illustration of Emotion-Focused Therapy. Available at: https://www.cpcab.co.uk/shop/transforming-emotional-pain-video.

[ref24] Crangle C. E. Wang R. Perreau-Guimaraes M. Nguyen M. U. Nguyen D. T. Suppes P. (2019). Machine learning for the recognition of emotion in the speech of couples in psychotherapy using the Stanford Suppes brain lab psychotherapy dataset. Available at: http://arxiv.org/abs/1901.04110

[ref25] Dasgupta P. B. (2017). Detection and analysis of human emotions through voice and speech pattern processing. Int. J. Comp. Trends Technol. 52, 1–3. doi: 10.14445/22312803/ijctt-v52p101

[ref26] Diamond G. M. Rochman D. Amir O. (2010). Arousing primary vulnerable emotions in the context of unresolved anger: “speaking about” versus “speaking to”. J. Couns. Psychol. 57, 402–410. doi: 10.1037/a0021115

[ref27] Gilbert P. Clarke M. Hempel S. Miles J. Irons C. (2004). Criticizing and reassuring oneself: an exploration of forms, styles and reasons in female students. Br. J. Clin. Psychol. 43, 31–50. doi: 10.1348/014466504772812959, PMID: 15005905

[ref28] Goldman R. N. (2017). Case formulation in emotion-focused therapy. Person-Center. Exp. Psychother. 16, 88–105. doi: 10.1080/14779757.2017.1330705

[ref29] Greenberg L. (2015). Emotion-focused therapy: Coaching clients to work through their feelings (2nd). Washington: American Psychological Association.

[ref30] Greenberg L. Malcolm W. (2002). Resolving unfinished business: relating process to outcome. J. Consult. Clin. Psychol. 70, 406–416. doi: 10.1037/0022-006X.70.2.406, PMID: 11952199

[ref31] Greenberg L. Watson J. C. (2006). Emotion-focused therapy for depression. Washington: American Psychological Association

[ref32] Haberman A. Shahar B. Bar-Kalifa E. Zilcha-Mano S. Diamond G. M. (2018). Exploring the process of change in emotion-focused therapy for social anxiety. Psychother. Res. 29, 908–918. doi: 10.1080/10503307.2018.1426896, PMID: 29366385

[ref33] Halamová J. Kanovský M. (2019). Emotion-focused training for emotion coaching – an intervention to reduce self-criticism. Hum. Aff. 29, 20–31. doi: 10.1515/humaff-2019-0003

[ref34] Halamová J. Kanovský M. Varšová K. Kupeli N. (2018). Randomised controlled trial of the new short-term online emotion focused training for self-compassion and self-protection in a nonclinical sample. Curr. Psychol. 40, 333–343. doi: 10.1007/s12144-018-9933-4, PMID: 33488040 PMC7799374

[ref35] Halamová J. Koróniová J. Kanovský M. Kénesy Túniyová M. Kupeli N. (2019). Psychological and physiological effects of emotion-focused training for self-compassion and self-protection. Res. Psychother. Psychopathol. Process Outcome 22, 264–279. doi: 10.4081/ripppo.2019.358, PMID: 32913797 PMC7451316

[ref36] Herrmann I. R. Greenberg L. Auszra L. (2016). Emotion categories and patterns of change in experiential therapy for depression. Psychother. Res. 26, 178–195. doi: 10.1080/10503307.2014.958597, PMID: 25265453

[ref37] Kamiloğlu R. G. Fischer A. H. Sauter D. A. (2020). Good vibrations: a review of vocal expressions of positive emotions. Psychon. Bull. Rev. 27, 237–265. doi: 10.3758/s13423-019-01701-x, PMID: 31898261 PMC7093353

[ref38] Kannan D. Levitt H. M. (2013). A review of client self-criticism in psychotherapy. J. Psychother. Integr. 23, 166–178. doi: 10.1037/a0032355

[ref39] Kapoor K. Sagar Verma K. (2019). Feature extraction in emotion recognition: an analysis of emotion using Praat. Int. J. Adv. Res. 5, 1881–1884.

[ref40] Kappas A. Hess U. Scherer K. R. (1991). “Voice and emotion” in Fundamentals of nonverbal behavior. ed. Rime R. F. B. (Cambridge: Cambridge University Press Katsikitis)

[ref41] Kramer U. Pascual-Leone A. (2015). The role of maladaptive anger in self-criticism: a quasi-experimental study on emotional processes. Couns. Psychol. Q. 29, 311–333. doi: 10.1080/09515070.2015.1090395

[ref42] Kumar A. Iqbal J. L. M. (2019). Machine learning based emotion recognition using speech signal. Int. J. Eng. Adv. Technol. 9, 295–302. doi: 10.35940/ijeat.a1068.1291s52019

[ref43] Luyten P. Blatt S. J. (2012). Psychodynamic treatment of depression. Psychiatr. Clin. N. Am. 35, 111–129. doi: 10.1016/j.psc.2012.01.001 22370494

[ref44] Magdin M. Sulka T. Tomanová J. Vozár M. (2019). Voice analysis using Praat software and classification of user emotional state. Int. J. Interact. Multim. Artif. Intell. 5, 33–42. doi: 10.9781/ijimai.2019.03.004

[ref45] Murray I. R. Arnott J. L. (1993). Toward the simulation of emotion in synthetic speech: a review of the literature on human vocal emotion. J. Acoust. Soc. Am. 93, 1097–1108. doi: 10.1121/1.405558, PMID: 8445120

[ref46] Nardone S. Pascual-leone A. Kramer U. Nardone S. (2021). “Strike while the iron is hot”: increased arousal anticipates unmet needs. Couns. Psychol. Q. 35, 110–128. doi: 10.1080/09515070.2021.1955659

[ref47] Neff K. D. (2003). The development and validation of a scale to measure self-compassion. Self Identity 2, 223–250. doi: 10.1080/15298860309027

[ref48] Parsa V. Jamieson D. G. (2001). Acoustic discrimination of pathological voice: sustained vowels versus continuous speech. J. Speech Lang. Hear. Res. 44, 327–339. doi: 10.1044/1092-4388(2001/027) 11324655

[ref49] Pascual-Leone A. (2017). How clients “change emotion with emotion”: a programme of research on emotional processing. Psychother. Res. 28, 165–182. doi: 10.1080/10503307.2017.1349350, PMID: 28714778

[ref50] Pascual-Leone A. Gilles P. Singh T. Andreescu C. A. (2013). Problem anger in psychotherapy: an emotion-focused perspective on hate, rage, and rejecting anger. J. Contemp. Psychother. 43, 83–92. doi: 10.1007/s10879-012-9214-8

[ref51] Pascual-Leone A. Greenberg L. (2005). “Classification of affective-meaning states (CAMS)” in Emotional processing in the therapeutic hour: Why “the only way out is through” Unpublished doctoral thesis. ed. Pascual-Leone A. (Toronto: York University)

[ref52] Pascual-Leone A. Greenberg L. (2007). Emotional processing in experiential therapy: why “the only way out is through.”. J. Consult. Clin. Psychol. 75, 875–887. doi: 10.1037/0022-006X.75.6.875 18085905

[ref53] Pascual-Leone A. Paivio S. C. (2013). “Emotion-focused therapy for anger in complex trauma” in Treatments for anger in specific populations: Theory, application, and outcome. ed. Fernandez E. (Oxford: Oxford University Press)

[ref54] Psychological and Educational Films. (1989). Integrative psychotherapy a six part series. Part 5: A demonstration with Dr. Leslie Greenberg. Corona Del Mar, CA: Psychological & Educational Films.

[ref55] R Core Team (2019). Audacity (2.3.3). Available at: https://www.audacityteam.org

[ref56] Rabiei M. Gasparetto A. (2015). A methodology for recognition of emotions based on speech analysis, for applications to human-robot interaction. An exploratory study Paladyn. J. Behav. Robot. 5, 1–11. doi: 10.2478/pjbr-2014-0001

[ref57] Razak A. A. Abidin M. I. Z. Komiya R. (2003). Emotion pitch variation analysis in Malay and English voice samples. 9th Asia-Pacific Conference on Communications (IEEE cat. No.03EX732).

[ref58] Rice L. N. Kerr G. P. (1986). “Measures of clients and therapist vocal quality” in The psychotherapeutic process: a research handbook. eds. Greenberg L. Pinshof W. M. (New York: Guilford Press)

[ref59] Rochman D. (2008). Appendix to: unresolved anger and sadness: identifying vocal acoustical correlates. J. Couns. Psychol. 55, 96–105. 10.1037/a0013720 22017557

[ref60] Rochman D. Diamond G. M. Amir O. (2008). Unresolved anger and sadness: identifying vocal acoustical correlates. J. Couns. Psychol. 55, 505–517. doi: 10.1037/a0013720, PMID: 22017557

[ref61] Sauter D. A. (2017). The nonverbal communication of positive emotions: an emotion family approach. Emot. Rev. 9, 222–234. doi: 10.1177/1754073916667236, PMID: 28804510 PMC5542129

[ref62] Scherer K. R. (2003). Vocal communication of emotion: a review of research paradigms. Speech Comm. 40, 227–256. doi: 10.1016/S0167-6393(02)00084-5

[ref63] Scherer K. R. Banse R. Wallbott H. G. Goldbeck T. (1991). Vocal cues in emotion encoding and decoding. Motiv. Emot. 15, 123–148. doi: 10.1007/BF00995674

[ref64] Scherer K. R. Clark-Polner E. Mortillaro M. (2011). In the eye of the beholder? Universality and cultural specificity in the expression and perception of emotion. Int. J. Psychol. 46, 401–435. doi: 10.1080/00207594.2011.626049, PMID: 22126090

[ref65] Scherer K. R. Johnstone T. Klasmeyer G. (2003). “Vocal expression of emotion” in Handbook of affective sciences. eds. Davidson R. J. Scherer K. R. Goldsmith H. H. (Oxford: Oxford University Press)

[ref66] Schröder M. Cowie R. Douglas-Cowie E. Westerdijk M. Gielen S. (2001). Acoustic correlates of emotion dimensions in view of speech synthesis. Eur. Secur. 2001, 87–90.

[ref67] Shahar B. Carlin E. R. Engle D. E. Hegde J. Szepsenwol O. Arkowitz H. (2012). A pilot investigation of emotion-focused two-chair dialogue intervention for self-criticism. Clin. Psychol. Psychother. 19, 496–507. doi: 10.1002/cpp.762, PMID: 21710579

[ref68] Simon-Thomas E. R. Keltner D. J. Sauter D. Sinicropi-Yao L. Abramson A. (2009). The voice conveys specific emotions: evidence from vocal burst displays. Emotion 9, 838–846. doi: 10.1037/a0017810, PMID: 20001126

[ref69] Singh T. Pascual-leone A. Morrison O. Greenberg L. (2020). Working with emotion predicts sudden gains during experiential therapy for depression. Psychother. Res. 31, 895–908. doi: 10.1080/10503307.2020.1866784, PMID: 33377419

[ref70] Sobin C. Alpert M. (1999). Emotion in speech: the acoustic attributes of fear, anger, sadness, and joy. J. Psycholinguist. Res. 28, 347–365. doi: 10.1023/A:1023237014909, PMID: 10380660

[ref71] Strauss C. Lever Taylor B. Gu J. Kuyken W. Baer R. Jones F.. (2016). What is compassion and how can we measure it? A review of definitions and measures. Clin. Psychol. Rev. 47, 15–27. doi: 10.1016/j.cpr.2016.05.004 27267346

[ref72] Timulak L. (2015). “Transforming emotional pain in psychotherapy” in An emotion-focused approach in transforming emotional pain in Psychotherapy (London: Routledge)

[ref73] Tomicic A. Martínez Guzmán C. (2011). Voice and psychotherapy: introduction to a line of research on mutual regultaion in psychotherapeutic dialog. PRAXIS. Revista de Psicología 13, 109–139.

[ref74] Traunmüller H. Eriksson A. (1994). The frequency range of the voice fundamental in the speech of male and female adults. Stockholm, Department of Linguistics, University of Stockholm.

[ref75] VideoLan. (2006). VLC media player (3.0.12). Available at: https://www.videolan.org/vlc/index.html

[ref76] Vogt T. André E. Bee N. (2008). EmoVoice - a framework for online recognition of emotions from voice. Percept. Multim. Dial. Syst. 5078, 188–199. doi: 10.1007/978-3-540-69369-7_21

[ref77] Waksler R. (2001). Pitch range and women’s sexual orientation. Word 52, 69–77. doi: 10.1080/00437956.2001.11432508

[ref78] Watson J. C. Bedard D. L. (2006). Clients’ emotional processing in psychotherapy: a comparison between cognitive-behavioral and process-experiential therapies. J. Consult. Clin. Psychol. 74, 152–159. doi: 10.1037/0022-006X.74.1.152, PMID: 16551152

[ref79] Whelton W. J. Greenberg L. (2005). Emotion in self-criticism. Personal. Individ. Differ. 38, 1583–1595. doi: 10.1016/j.paid.2004.09.024

[ref80] Whelton W. J. Henkelman J. J. (2002). A verbal analysis of forms of self-criticism. Alberta J. Educ. Res. 48, 88–90.

[ref81] Yildirim S. Bulut M. Lee C. M. Kazemzadeh A. Busso C. Deng Z.. (2004). An acoustic study of emotions expressed in speech. 8th International Conference on Spoken Language Processing.

